# Sex- and Dose-Dependent Effect of L-Citrulline on Body Weight and Food Intake in Obese Type 2 Diabetic Rats

**DOI:** 10.5812/ijem-162367

**Published:** 2025-10-20

**Authors:** Fatemeh Bagheripour, Sajad Jeddi, Asghar Ghasemi

**Affiliations:** 1 Endocrine Physiology Research Center, Research Institute for Endocrine Molecular Biology, Research Institute for Endocrine Sciences, Shahid Beheshti University of Medical Sciences, Tehran, Iran

**Keywords:** L-Citrulline, Body Weight, Food Intake, Water Consumption, Sex Differences, Rat

## Abstract

**Background:**

Obesity and type 2 diabetes (T2D) are associated with nitric oxide (NO) deficiency. L-citrulline (Cit), a substrate for NO synthesis, has been suggested as a treatment for obesity and T2D.

**Objectives:**

This study aims to determine the effects of Cit on body weight, food intake, and water consumption in obese T2D male and female rats.

**Methods:**

The T2D was induced using a high-fat diet (HFD) and a low dose of streptozotocin (STZ). Obese male (n = 30) and female (n = 30) rats with T2D were divided into five groups (n = 6/group) that received Cit (0, 1, 4, 7, and 10 g/L in drinking water for 8 weeks). Body weight, food intake, and water consumption were measured every week. Serum Cit and nitrite+nitrate (NOx) concentrations were measured at weeks 0, 4, and 8, and serum fasting glucose was measured at week 8.

**Results:**

Compared to non-treated T2D rats, Cit-treated male rats had lower body weight (11.3%, 13.0%, and 11.6% at doses of 4, 7, and 10 g/L), lower food intake (4.7% and 5.5% at doses of 4 and 7 g/L), and water consumption (7% at dose 7 g/L). In female rats, Cit decreased body weight (7.2%, 8%, and 7.3% at doses of 4, 7, and 10 g/L), food intake (4.0% and 5.0% at doses of 4 and 7 g/L), and water consumption (5% and 6.6% at doses of 4 and 7 g/L; all P < 0.001). The body weight and food intake-lowering effects of Cit were higher in T2D male rats than females (all P < 0.05), but decreased water consumption was comparable between sexes. A Cit dose of 7 g/L was most effective in reducing all measured parameters in both sexes.

**Conclusions:**

The Cit decreased body weight, food intake, and water consumption in obese T2D male and female rats. These effects were sex- and dose-dependent.

## 1. Background

The worldwide prevalence of obesity was 14% in 2020 and is anticipated to rise to 24% by 2035, affecting nearly 2 billion adults and children (1). Obesity is a significant risk factor for the progression of type 2 diabetes (T2D) (2), with approximately 44% of obese people developing T2D, and 80% of T2D patients being overweight or obese (3, 4). Apart from semaglutide, which leads to approximately 15% weight loss, other FDA-approved anti-obesity medications like orlistat, phentermine/topiramate, naltrexone/bupropion, and liraglutide typically result in only 3 - 10% weight loss, and these drugs have side effects such as the exacerbation of anxiety or depression (5, 6). Therefore, further research is necessary to identify more effective therapies for obesity and T2D.

L-citrulline (Cit), an amino acid found in high quantities in watermelon (7), is endogenously produced by the nitric oxide (NO) synthase (NOS) enzyme, which converts L-arginine (Arg) to NO and Cit (8). The NO bioavailability is decreased in animal models of obesity and diabetes (9, 10) and in humans with obesity (11) and T2D (12). The Cit is a precursor for synthesizing Arg and NO, suggesting its potential to enhance NO production in obesity and T2D (8). Administration of Cit at different doses ranging from 39 to 1000 mg/kg/day for durations of 4 (13), 8 (14), 9 (15, 16), 11 (15), and 12 (17) weeks decreases body weight (13-17) and food intake (15) in male rodents with obesity (15, 17) and T2D (13, 14).

To the best of our knowledge, studies investigating the body weight-lowering effects of Cit have been conducted in male rodents and have typically utilized a single dose (13, 15-20). Notably, one study from our lab has demonstrated the body weight-lowering effects of a single dose of Cit in both male and female rats (14). The effect of Cit in reducing fasting glucose, improving lipid profile, and enhancing glucose tolerance is higher in female rats with T2D than in male rats (14). In addition, the anti-diabetic and anti-dyslipidemic effects of Cit are dose-dependent in diabetic male rats (21).

## 2. Objectives

This study was designed to investigate the sex- and dose-dependent effects of Cit on body weight, food intake, and water consumption in rats with T2D.

## 3. Methods

### 3.1. Animals

This study used female (body weight = 170 - 180 g, 8-week-old) and male (body weight = 190 - 210 g, 8-week-old) Wistar rats. Rats were kept in a relatively constant temperature (22 ± 2°C) with a 12-hour light-dark cycle (lights on 07:00 - 19:00). They had unlimited access to tap water and a regular diet and were housed two per cage. All experiments were conducted in accordance with the published guidelines for the care and use of laboratory animals in Iran (22) and are reported according to the ARRIVE guidelines (23). The Research Institute for Endocrine Sciences Ethics Committee, affiliated with Shahid Beheshti University of Medical Sciences, confirmed and approved the proposal of the current study (IR.SBMU.ENDOCRINE.REC.1402.074).

### 3.2. Induction of Type 2 Diabetes

The T2D was induced by combining a high-fat diet (HFD) with a low dose of streptozotocin (STZ) (24). In this T2D model, insulin resistance develops three weeks after the consumption of an HFD, and following a low-dose injection of STZ, partial destruction of β-cells occurs (24). To prepare the 1000 g of HFD, the following was mixed: 586 g of regular powdered diet, 310 g of sheep butter as a source of fat, 73 g of casein as a source of protein, 1.8 g of DL-methionine, 4.1 g of a vitamin mix, and 25 g of a mineral mix. In the HFD, the total caloric content was 4900 kcal/kg, with 58.8% from fat, 27.0% from carbohydrates, and 14.2% from protein. In comparison, the regular diet had 3160 kcal/kg, with 5.7% from fat, 72.2% from carbohydrates, and 22.1% from protein (25).

Rats were maintained on an HFD for three weeks, and then STZ (30 mg/kg, dissolved in 0.1 mM citrate buffer, pH 4.5) was injected intraperitoneally into 12-hour fasted rats (fasting time 8:00 PM - 8:00 AM). One week after the STZ injection, blood samples were collected from the tip of the rat's tail under isoflurane anesthesia to confirm T2D. Rats with fasting serum glucose levels ranging from 150 to 350 mg/dL were classified as having T2D; following randomization into the Cit-treated groups, these rats were maintained on the HFD for the duration of the study (24).

### 3.3. Study Design

**Figure 1.**
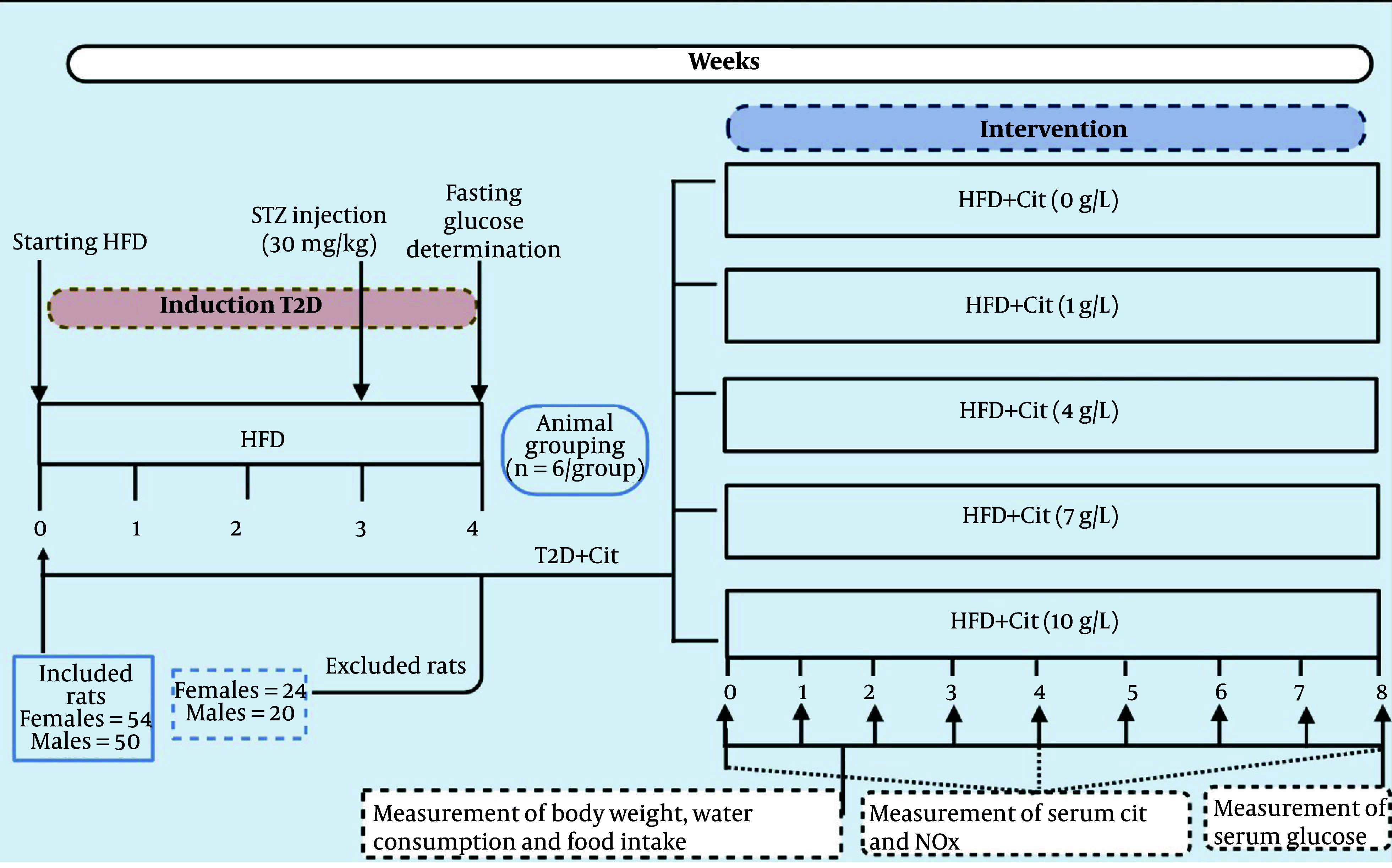
The study design (abbreviations: Cit, L-citrulline; HFD, high-fat diet; NOx, nitrite+nitrate; STZ, streptozotocin; T2D, type 2 diabetes).

Figure 1 illustrates the procedure of this experimental study. A total of 104 rats, consisting of 54 females and 50 males, were included in this study. Based on our prior experience, the achievement percentage of T2D induction in our laboratory was approximately 60% (26). Of the 54 female rats given the HFD and low-dose STZ, 24 were excluded — 11 had fasting glucose levels below 150 mg/dL, and 13 above 350 mg/dL. The remaining 30 rats, with glucose levels between 150 and 350 mg/dL, were classified as diabetic. Of the 50 male rats given the HFD and low-dose STZ, 20 were excluded — 11 had fasting glucose levels below 150 mg/dL, and 9 above 350 mg/dL. The remaining 30 rats, with glucose levels between 150 and 350 mg/dL, were classified as diabetic. Hence, the achievement percentage for T2D induction was 56% in female and 60% in male rats.

After verifying the induction of the T2D model, female and male T2D rats were randomly (Appendix 1 in Supplementary File) divided into 5 groups (n = 6/group) that received Cit at doses of 0, 1, 4, 7, and 10 g/L in drinking water for 8 weeks, respectively. The rationale behind selecting these doses was that Cit has been used in doses ranging from 2.7 to 15 g/day (40 - 215 mg/kg/day in a 70-kg human) in humans (8), and it has been proposed that doses of 3 - 10 g/day (43 - 143 mg/kg/day in a 70-kg human) are most effective for cardiometabolic disorders (27). When translated to rats, the doses of 43 - 143 mg/kg/day in humans (rat dose = human dose/0.162) (28), yielded doses of 265 and 882 mg/kg in rats, respectively. Considering the initial body weight of animals (~ 250 g) and the average value of water intake in rats (30 mL/day) (29), doses of Cit that have been used in this study (1, 4, 7, and 10 g/L in drinking water) correspond to 120, 480, 840, and 1200 mg/kg, which cover the intended doses.

Body weight (using Tefal scale; sensitivity 1 g), food intake (using Perfect scale; sensitivity 1 g), and water consumption (using a graduated cylinder) were measured every week during the experimental period. Mean food consumption (g/100 g body weight) and water consumption (mL/100 g body weight) were determined at 1-week intervals; the amount of food and water consumed per cage was divided by the number of rats housed in the cage (2 rats). The amount of food and water consumed per cage during these time intervals was calculated by subtracting the residual food and water recovered from each cage from the total amount of food (300 g) and water (1000 mL) presented.

Fasted (12 h) blood samples were collected from rats' tails at weeks 0, 4, and 8 to measure serum Cit and nitrite+nitrate (NOx) and fasting serum glucose at week 8. Blood samples were centrifuged using a refrigerated centrifuge (Hettich Company, Tuttlingen, Germany) at 5,000 g for 10 minutes to separate sera. Serum glucose (using the glucose oxidase method) was measured using a commercially available kit (ParsAzmoon Company, Tehran, Iran) by an autoanalyzer (Selectra E, serial number 0-2124, Netherlands). Intra- and inter-assay CVs were 2.3% and 2.0%, respectively. At the end of the study (at week 8), rats were anesthetized using sodium pentobarbital (60 mg/kg) and euthanized by exsanguination through left ventricular cardiac puncture.

### 3.4. Measurement of L-Citrulline

The Cit concentration was measured using a colorimetric method (30). To eliminate urea interference, 200 μL of urease solution (6 U) was added to 200 μL of serum and incubated for 60 minutes at 37°C. Then, 60 μL of treated serum was mixed with 200 μL of coloring solution; the coloring solution was 1 part of solution A [diacetyl monoxime (DAMO), 80 mM and thiosemicarbazide (TSC) 2 mM] and 3 parts of solution B [phosphoric acid, 17%, sulfuric acid, 34%, and iron (Ш) chloride (FeCl_3_), 1.5 mM]. After 15 minutes of incubation at 95°C and 10 minutes at room temperature, absorbance was measured at 540 nm using an ELISA reader (BioTek, Power wave XS2, USA). The Cit levels were calculated using a 0 - 200 μmol/L Cit standard curve (Appendix 2 in Supplementary File). Intra- and inter-assay CVs were 3.8% and 4.4%, respectively.

### 3.5. Measurement of Nitrite+Nitrate

Serum NOx levels were measured using a modified Griess method (31). Samples were deproteinized with zinc sulfate (15 mg/mL) (32), and NaOH (3.72 M) (33) was added to decrease the turbidity of the samples, then centrifuged (10 minutes, 10,000 g). Supernatant (100 μL) was mixed with vanadium (III) chloride (100 μL, 8 mg/mL), sulfanilamide (50 μL, 2% in 5% HCl), and N-(1-Naphtyl) ethylenediamine dihydrochloride (NEDD) (50 μL, 0.1% in ddH_2_O). After 30 minutes of incubation at 37°C, absorbance was read at 540 nm using an ELISA. The NOx concentrations were calculated using a 0 - 100 μmol/L sodium nitrate standard curve. Intra- and inter-assay CVs were 2.3% and 3.4%, respectively.

### 3.6. Statistical Analyses

Statistical analyses were performed using GraphPad Prism software version 8.00 (GraphPad Software, La Jolla, California USA, www.Graphpad.com). Data are reported as mean ± standard error of the mean (SEM). Two-way mixed (between-within) analysis of variance (ANOVA) followed by the Bonferroni post-hoc test was used to analyze data on body weight, food intake, and water consumption, as well as serum levels of Cit and NOx over time for either sex. One-way ANOVA followed by the Bonferroni post-hoc test was used in either sex for comparing the area under the curves (AUCs) of body weight, food intake, water consumption, serum levels of Cit and NOx over 8 weeks of intervention, as well as serum glucose at the end of the study. The student’s *t*-test was used to compare the percentage of body weight, food intake, water consumption, and serum levels of Cit and NOx changes between males and females. Two-sided P < 0.05 was considered statistically significant.

## 4. Results

### 4.1. Effect of L-Citrulline on Serum Glucose, L-Citrulline, and Nitrite+Nitrate in Male Rats

**Figure 2.**
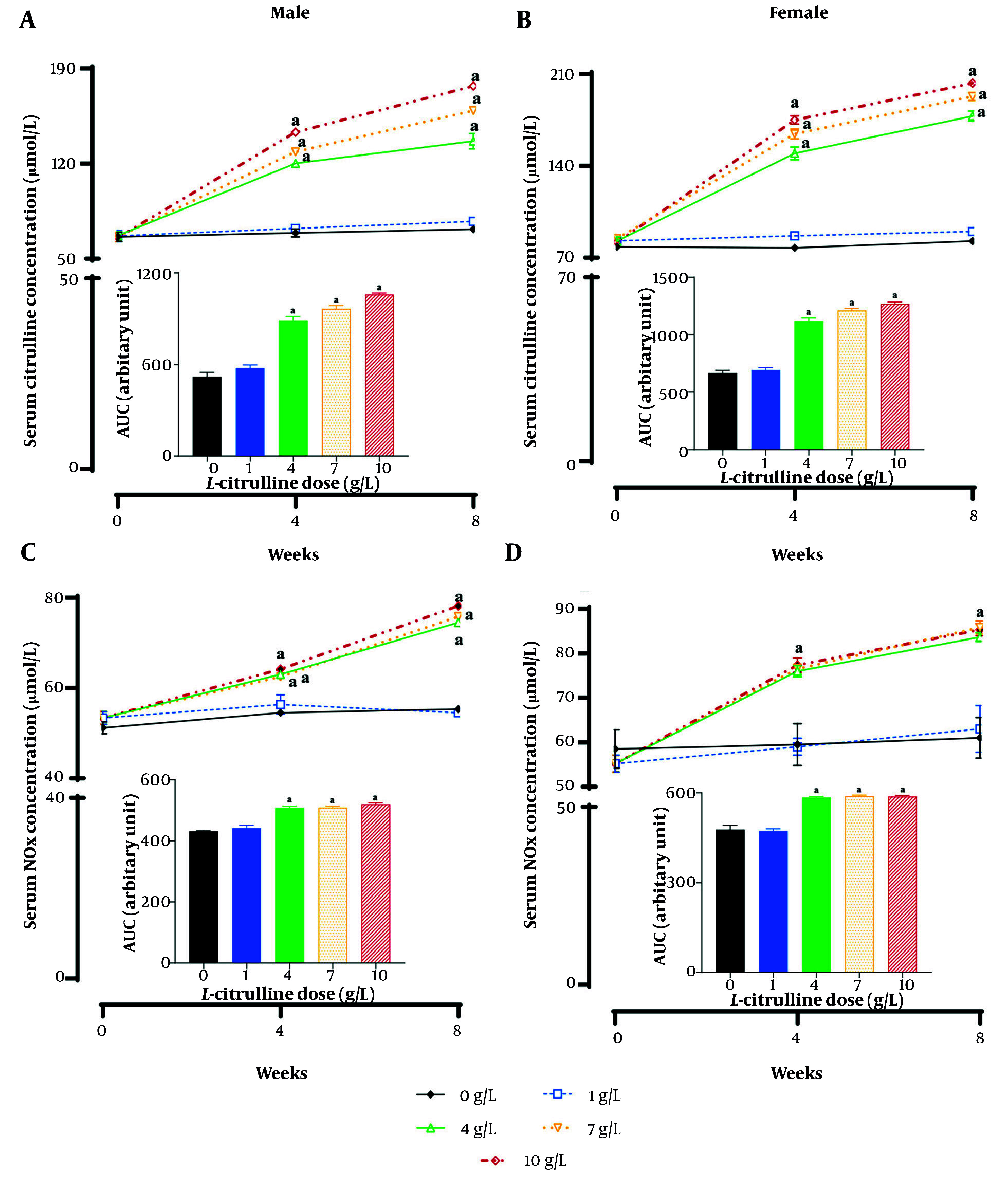
Effect of different doses of L-citrulline (Cit) on A and B, the serum Cit and nitrite+nitrate (NOx, C and D) levels in male and female rats with type 2 diabetes [T2D; values are mean ± standard error of the mean (SEM); n = 6/group; a, statistically significant difference in comparison to the dose of 0 g/L Cit; abbreviation: AUC, area under the curve].

Compared with non-treated T2D male rats (233.0 ± 1.9 mg/dL), Cit-treated T2D male rats had lower fasting glucose at the end of the study at Cit doses of 4 (206.8 ± 1.8 mg/dL), 7 (207.5 ± 0.9 mg/dL), and 10 (210.0 ± 0.8 mg/dL) g/L, respectively (all P < 0.001). As shown in Figure 2A, compared to non-treated T2D male rats (64.1 ± 3.4 µmol/L), Cit-treated T2D male rats had higher serum Cit levels at doses of 4 (120.2 ± 2.6 µmol/L), 7 (128.7 ± 2.9 µmol/L), and 10 g/L (143.2 ± 1.5 µmol/L) Cit, at week 4 (all P < 0.001). A similar pattern of increases in serum Cit levels was observed at doses of 4 (136.6 ± 5.7 µmol/L), 7 (158.9 ± 2.9 µmol/L), and 10 g/L (177.2 ± 0.9 µmol/L) Cit, compared to non-treated (67.7 ± 4.2 µmol/L) T2D male rats, at the end of the study (week 8) (all P < 0.001). The AUCs showed that Cit-treated T2D male rats, compared with non-treated T2D male ones, had higher Cit levels at doses of 4 (71%), 7 (86%), and 10 g/L (96%) Cit over the 8-week intervention (all P < 0.001).

Compared to non-treated (54.5 ± 0.4 µmol/L) T2D male rats, Cit administration significantly increased serum NOx levels in Cit-treated T2D male rats at doses of 4 (63.0 ± 0.9 µmol/L), 7 (63.5 ± 0.8 µmol/L), and 10 g/L (64.2 ± 0.6 µmol/L) Cit, at week 4 (all P < 0.001). The Cit administration increased serum NOx levels at doses of 4 (74.5 ± 0.8 µmol/L), 7 (75.8 ± 0.7 µmol/L), and 10 g/L (78.2 ± 0.5 µmol/L) Cit, compared to non-treated (55.3 ± 0.3 µmol/L) T2D male rats, at the end of the study (week 8; all P < 0.001, Figure 2C). The AUCs showed that Cit-treated T2D male rats compared with non-treated T2D male ones had higher NOx levels at doses of 4 (19%), 7 (19%), and 10 g/L (21%) Cit over the 8-week intervention (all P < 0.001). The Cit administration at a dose of 1 g/L did not affect fasting glucose, Cit, and NOx levels in T2D male rats.

### 4.2. Effect of L-Citrulline on Body Weight, Food Intake, and Water Consumption in Male Rats

**Figure 3.**
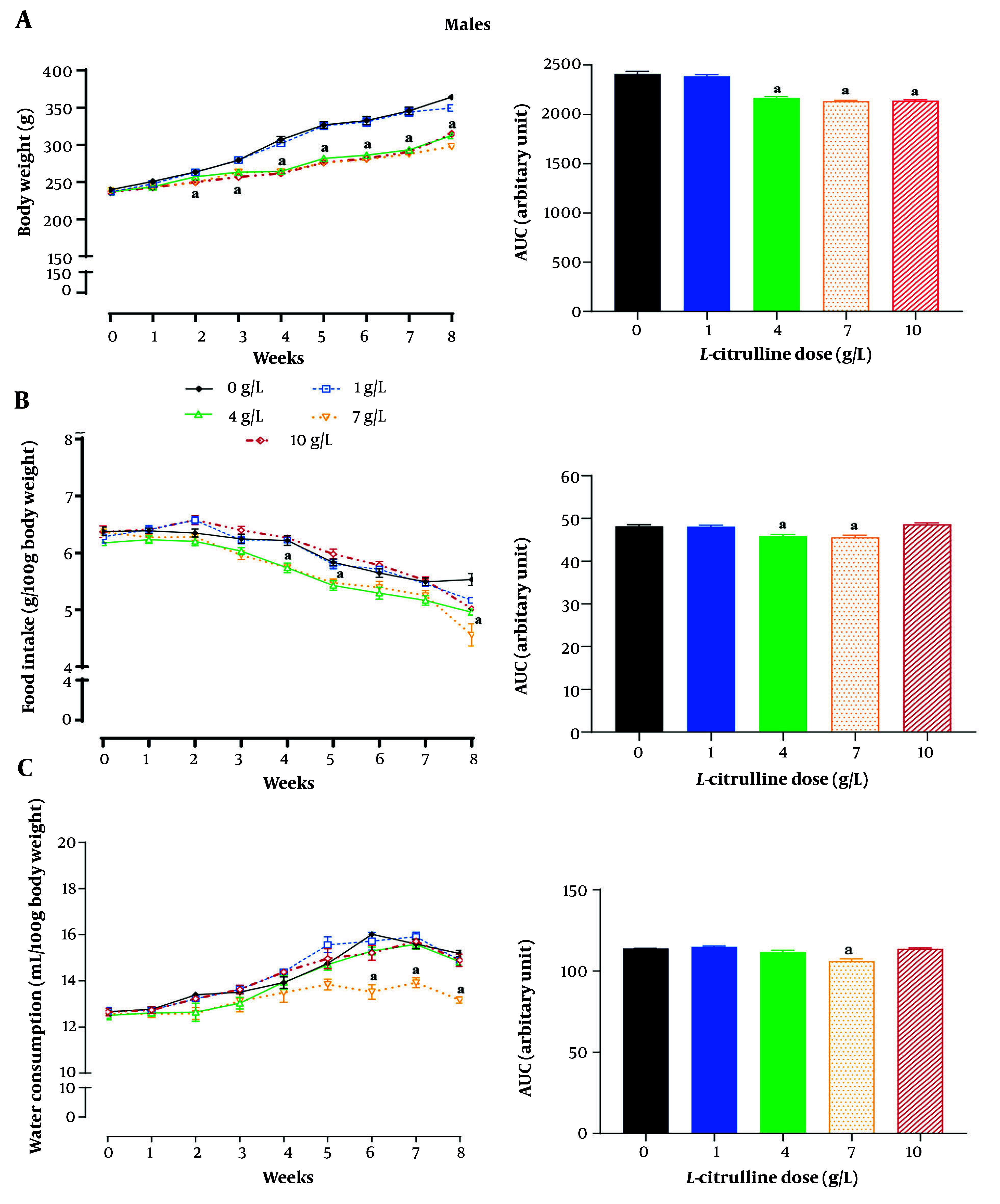
Effect of different doses of L-citrulline (Cit) on A, body weight; B, food intake; and C, water consumption in male rats with type 2 diabetes [T2D; values are mean ± standard error of the mean (SEM); n = 6/group; a, statistically significant difference in comparison to the doses of 0 g/L Cit; abbreviation: AUC, area under the curve].

As shown in Figure 3A, compared to non-treated T2D male rats, Cit-treated T2D male rats displayed a decrease in body weight from week 2 at doses of 7 and 10 g/L and from week 3 at a dose of 4 g/L. The AUCs showed that Cit-treated T2D male rats, compared with non-treated T2D male ones, had lower body weights of 11.3%, 13.0%, and 11.6% at Cit doses of 4, 7, and 10 g/L, respectively (all P < 0.001) over the 8-week intervention.

Compared to non-treated T2D male rats, decreased food intake began at week 4 at doses of 4 and 7 g/L and at week 8 at a dose of 10 g/L of Cit (Figure 3B). The AUCs showed that Cit-treated T2D male rats, compared with non-treated T2D male ones, had lower food intake at doses of 4 (4.7%) and 7 g/L (5.5%) Cit (all P < 0.001).

Compared to non-treated T2D male rats, decreased water consumption began at week 6 at doses of 7 g/L of Cit (all P < 0.001). The Cit-treated T2D male rats, compared with non-treated T2D male ones, had lower water consumption of 7.0% at Cit doses of 7 g/L (as measured from AUCs; Figure 3C). 

**Figure 4.**
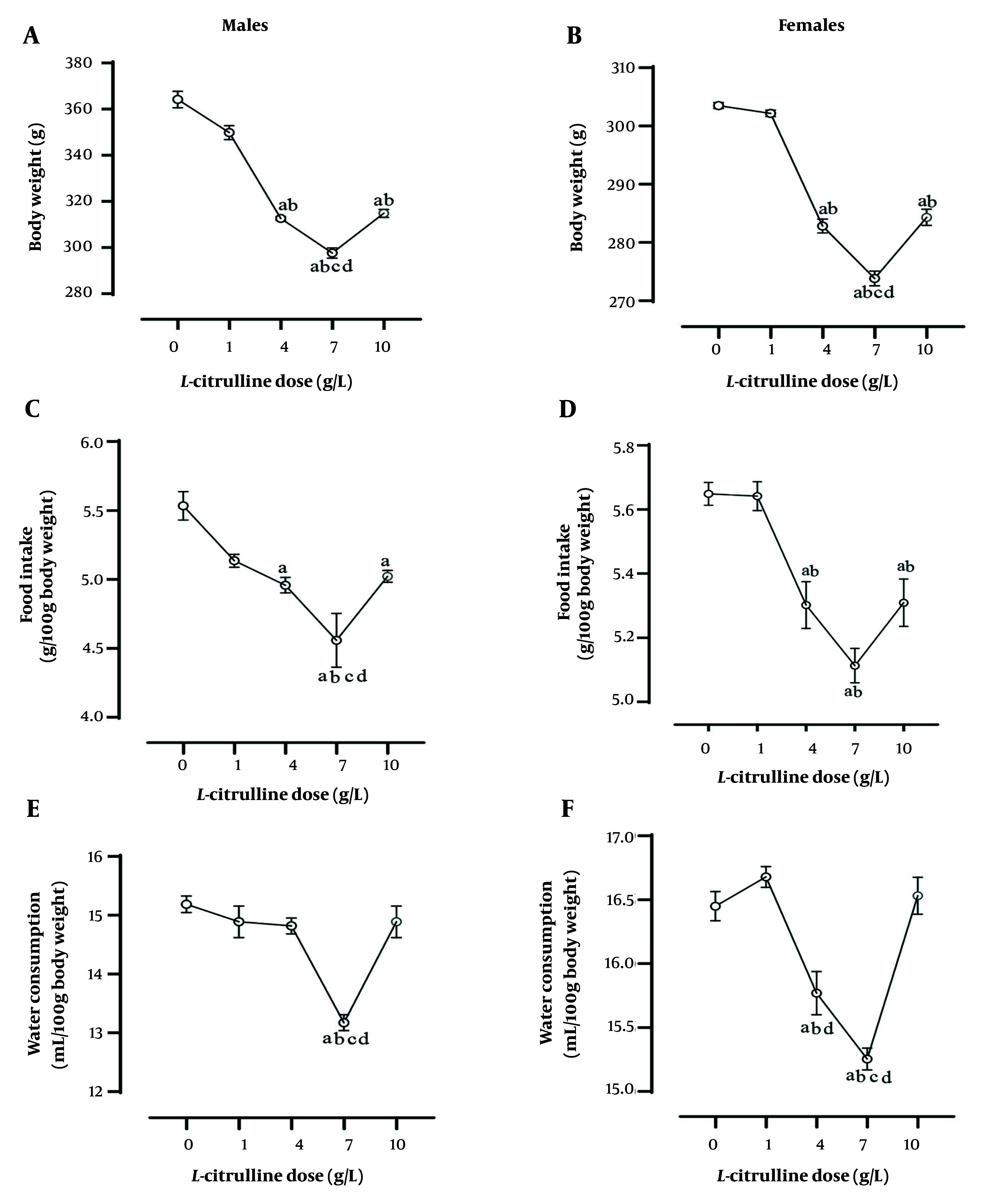
Dose-dependent effect of L-citrulline (Cit) on A and B, body weight; C and D, food intake; and E and F, water consumption at the end of the study (week 8) in male and female rats with type 2 diabetes [T2D; values are mean ± standard error of the mean (SEM); n = 6/group; a, b, c, and d, statistically significant differences in comparison to the doses of 0, 1, 4, and 10 g/L Cit, respectively].

The Cit administration at a dose of 1 g/L did not affect body weight and food intake, and at doses of 1, 4, and 10 g/L did not affect water consumption in T2D male rats. In addition, the most significant effect of Cit in decreasing body weight, food intake, and water consumption was seen at a dose of 7 g/L Cit (all P < 0.001; Figure 4A, C, and E).

### 4.3. Effect of L-Citrulline on Serum Glucose, L-Citrulline, and Nitrite+Nitrate Levels in Female Rats

Compared with non-treated T2D female rats (208.3 ± 2.1 mg/dL), Cit-treated T2D female rats had lower fasting glucose at the end of the study (week 8) at Cit doses of 4 (153.7 ± 0.9 mg/dL), 7 (154.7 ± 1.1 mg/dL), and 10 (155.7 ± 0.7 mg/dL) g/L, respectively (all P < 0.001).

As shown in Figure 2B, compared to non-treated (83.6 ± 3.5 µmol/L) T2D female rats, Cit-treated T2D female rats had higher serum Cit levels at doses of 4 (149.5 ± 4.8 µmol/L), 7 (164.3 ± 3.8 µmol/L), and 10 g/L (174.8 ± 3.2 µmol/L) Cit, at week 4 (all P < 0.001). A similar pattern of increase in serum Cit levels was observed at doses of 4 (177.8 ± 3.9 µmol/L), 7 (192.8 ± 3.2 µmol/L), and 10 g/L (202.9 ± 1.4 µmol/L) Cit, compared to non-treated (82.4 ± 2.8 µmol/L) T2D female rats, at the end of the study (week 8, all P < 0.001). The AUCs showed that Cit-treated T2D female rats, compared with non-treated T2D female ones, had higher Cit levels at doses of 4 (68%), 7 (82%), and 10 g/L (91%) Cit (all P < 0.001).

Compared to non-treated (59.5 ± 1.9 µmol/L) T2D female rats, Cit administration significantly increased serum NOx levels in Cit-treated T2D female rats at doses of 4 (76.0 ± 0.5 µmol/L), 7 (76.5 ± 0.4 µmol/L), and 10 g/L (77.3 ± 0.7 µmol/L) Cit, at week 4 (all P < 0.001). The Cit administration increased serum NOx levels at doses of 4 (83.7 ± 0.4 µmol/L), 7 (87.3 ± 0.3 µmol/L), and 10 g/L (87.2 ± 0.6 µmol/L), compared to non-treated T2D female rats (61.0 ± 1.9 µmol/L), at the end of the study (week 8; all P < 0.001, Figure 2D). The AUCs showed that Cit-treated T2D female rats compared with non-treated T2D female ones had higher NOx levels at doses of 4 (21%), 7 (22%), and 10 g/L (23%) Cit (all P < 0.001). The Cit administration at a dose of 1 g/L did not affect fasting glucose, Cit, and NOx levels in T2D female rats.

### 4.4. Effect of L-Citrulline on Body Weight, Food Intake, and Water Consumption in Female Rats

**Figure 5.**
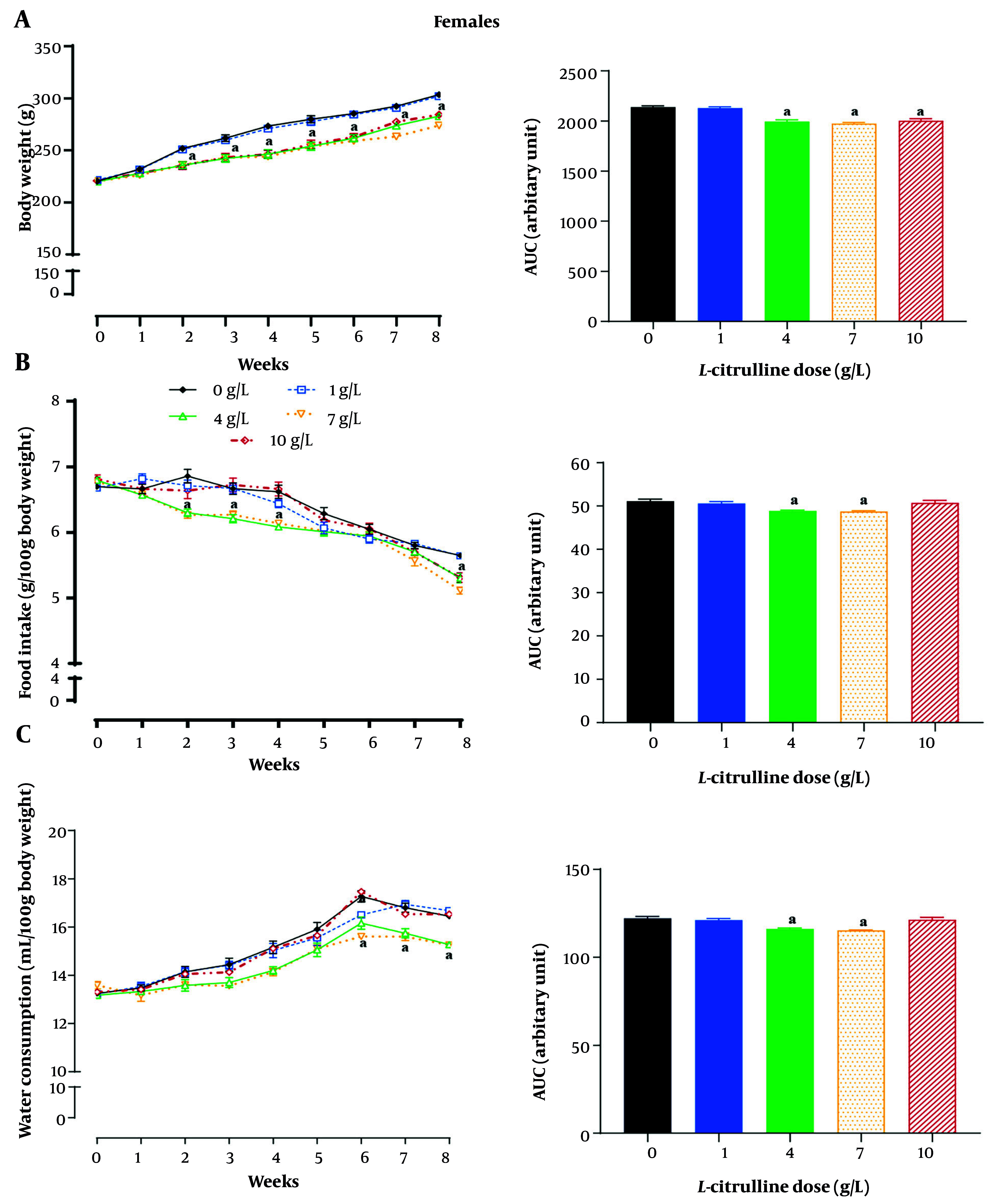
Effect of different doses of L-citrulline (Cit) on A, body weight; B, food intake; and C, water consumption in female rats with type 2 diabetes [T2D; values are mean ± standard error of the mean (SEM); n = 6/group; a, statistically significant difference in comparison to the doses of 0 g/L Cit; abbreviation: AUC, area under the curve].

As shown in Figure 5A, compared to non-treated T2D female rats, Cit-treated T2D female rats displayed a decrease in body weight from week 2 at doses of 4, 7, and 10 g/L Cit. The Cit-treated T2D female rats, compared with non-treated T2D female ones, had lower body weights of 7.2%, 8%, and 7.3% at Cit doses of 4, 7, and 10 g/L, respectively (all P < 0.001) over the 8-week intervention (as measured from AUCs).

Compared to non-treated T2D female rats, decreased food intake began at week 2 at doses of 4 and 7 g/L and at week 8 at a dose of 10 g/L (Figure 5B) of Cit. The AUCs showed that Cit-treated T2D female rats, compared with non-treated T2D female ones, had lower food intake at doses of 4 (4.0%) and 7 g/L (5.0%) Cit (all P < 0.001).

Compared to non-treated T2D female rats, decreased water consumption began at week 7 at a dose of 4 g/L and at week 6 at a dose of 7 g/L (Figure 5C) of Cit. The AUCs showed that Cit-treated T2D female rats, compared with non-treated T2D female ones, had lower water consumption at doses of 4 (5.0%) and 7 g/L (6.6%) Cit (all P < 0.001).

The Cit administration at a dose of 1 g/L did not affect body weight and food intake, and at doses of 1 and 10 g/L did not affect water consumption in T2D female rats. In addition, the greatest effect of Cit in decreasing body weight and water consumption was seen at a dose of 7 g/L Cit (all P < 0.001, Figure 4B - F).

### 4.5. Sex-Dependent Effect of L-Citrulline

**Figure 6.**
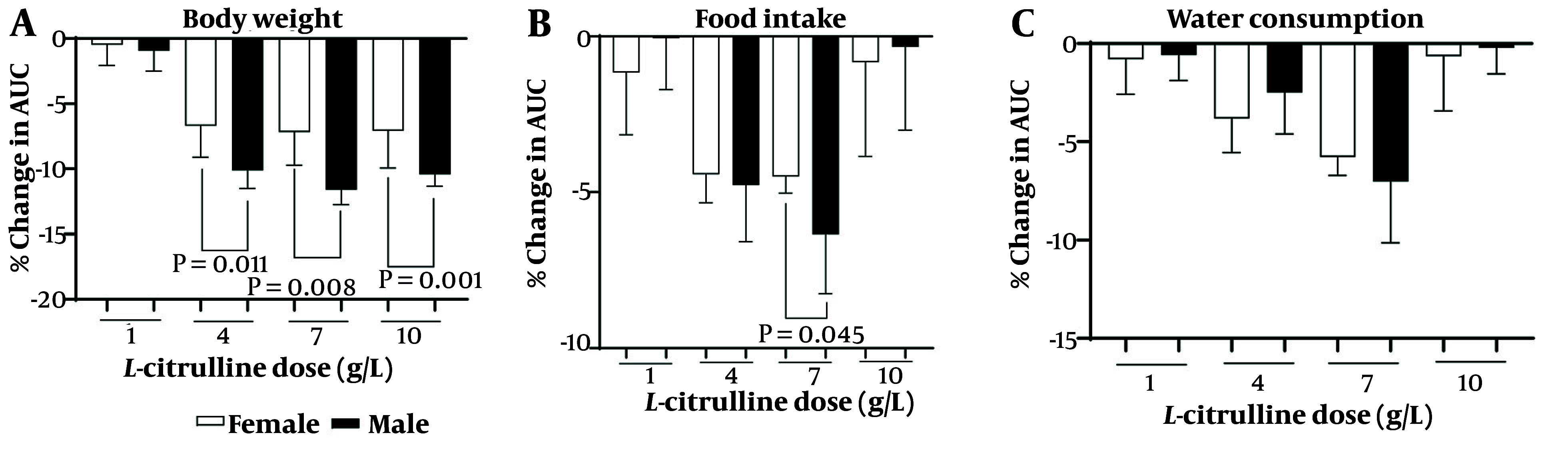
Sex difference in the effects of different doses of L-citrulline (Cit) on A, body weight; B, food intake; and C, water consumption in rats with T2D (Abbreviations: AUC, area under the curve; T2D, type 2 diabetes).

As shown in Figure 6A, the body weight-lowering effects of Cit were higher in male than female rats at doses of 4 (11.3% vs. 7.2%, P = 0.011), 7 (13.0% vs. 8%, P = 0.008), and 10 (11.6% vs. 7.3%, P = 0.001) g/L. Cit at a dose of 7 g/L (6.3% vs. 4.5%, P = 0.045) was more effective in decreasing food intake in male compared to female rats (Figure 6B). The effect of Cit on water consumption was not sex-dependent (Figure 6C). Increasing Cit levels in T2D rats following Cit administration at doses of 4 (71% vs. 68%), 7 (86% vs. 82%), and 10 (96% vs. 91%) g/L were comparable between male and female. The effect of Cit on increasing serum NOx at doses of 4 (19% vs. 21%), 7 (19% vs. 22%), and 10 (21% vs. 23%) g/L in T2D rats was comparable between male and female.

## 5. Discussion

The findings of this study showed that Cit reduces body weight, food intake, and water consumption in both male and female rats with T2D. These effects were sex-dependent (male > female; except for Cit effects on water consumption) and dose-dependent (best dose = 7 g/L). In our study, as expected, and as has been previously reported (13, 14, 34), Cit increased serum Cit and NOx concentrations in T2D rats. These findings indicate the effectiveness of our intervention.

Our results on the body weight and food intake-lowering effects of Cit in male rats are in line with previous studies in rodents (13-17), as summarized in Table 1. The Cit has been dosed in the range of 39 to 2000 mg/kg/day over a duration of 3 to 15 weeks (13-20). In our study, the doses of Cit that rats finally used according to their water consumption (170, 700, 1225, and 1750 mg/kg/day) and intervention period (8 weeks) are within these ranges. In addition, our results extrapolate previous findings by demonstrating a dose-dependent effect of Cit in decreasing body weight, food intake, and water consumption in T2D male rats.

**Table 1.** Summary of Animal Studies that Assessed Effects of L-Citrulline on Body Weight, Water Consumption, and Food Intake

Studies	Animal	Model	Sex	Age (wk)	Intervention	Route	Dose of Cit (mg/kg)	Duration (wk)	Body Weight	Water Consumption	Food Intake	Serum Cit	Serum NOx
**Poduri et al. (** 17 **)**	Mouse	LDL receptor deficient	Male	6	Watermelon	Drinking water	39 - 58	12	↓ (9%)	↔	↔	↑ (43%)	NR
**Capel et al. (** 18 **)**	Mouse	Obese	Male	5	Cit	Drinking water	2500	3	↔	NR	↔	↑ (71%)	NR
**Eshreif et al. (** 19 **)**	Mouse	Obese	Male	10	Cit	Drinking water	150	15	↔	NR	↔	NR	NR
**Kudo et al. (** 15 **)**	Mouse	Obese+hyperglycemia	Male	6	Cit	Drinking water	1000	9	↓ (17%)	NR	↓ (16%)	NR	NR
**Kudo et al. (** 15 **)**	Rat	Obese	Male	5	Cit	Drinking water	500	11	↓ (9%)	NR	↓ (11%)	NR	NR
**Wu et al. (** 13 **)**	Rat	T2D+obese	Male	8	Watermelon	Drinking water	600	4	↓ (%3)	↔	↔	↑ (141%)	↑ (41%)
**Yoshitomi et al. (** 20 **)**	Rat	Obese	Male	5	Cit	Drinking water	2000	8	↔	NR	↔	NR	NR
**Kudo et al. (** 16 **)**	Rat	Nonalcoholic fatty liver disease	Male	6	Cit	Gavage	500	9	↓ (7%)	NR	↔	NR	NR
**Bagheripour et al. (** 14 **)**	Rat	T2D+obese	Male and female	8	Cit	Drinking water	700	8	↓ (10% male and 7% female)	NR	NR	↑ (71% male and 68% female)	↑ (18% male and 23% female)

Abbreviations: Cit, L-citrulline; NOx, nitrite+nitrate; LDL, low-density lipoprotein; NR, not reported; T2D, type 2 diabetes.

The Cit is safe and well-tolerated at doses up to 15 g/day in humans (35) and 5.7 g/kg in animals (36). However, its high doses (15 g/day for 2 weeks) may be associated with side effects, including nausea, headache, lightheadedness, and diarrhea (37). In addition, Cit may interact with antihypertensive and antidiabetic drugs; for instance, metformin lowers plasma Cit levels in both diabetic patients and mice (38, 39).

In our study, Cit decreased food intake in female rats with T2D in a dose-dependent and sex-dependent manner. This finding is similar to that of Kudo et al. in obese male Sprague-Dawley rats and obese male kk-Ay mice (15). The Cit increases the expression of proopiomelanocortin (POMC), a peptide that suppresses food intake in the hypothalamus, thereby reducing food intake (15). In addition, Cit can enter the Cit-Arg cycle and increase NO production (8). In line with this notion, our observations revealed increased serum NOx levels in response to Cit treatment. Additionally, Arg, the precursor of NO, decreases appetitive behaviors, including sniffing and approaching food, and reduces the number of feeding bouts in rats (40).

We also showed that the food intake-lowering effects of Cit are dose-dependent, with a Cit dose of 1225 mg/kg having the greatest effect. Similarly, it has been shown that the improving effect of Cit on the lipid profile in T2D rats is dose-dependent, with the best response obtained at 400 mg/kg (21). In the current study, the effect of Cit in decreasing food intake was higher in male than female rats. Female rats have higher serum ghrelin than males (41). The stimulatory effect of ghrelin on food intake in rats is NO-dependent (42), and serum NOx is higher in T2D female rats compared to male ones (14). Thus, sex-dependent effects of Cit on food intake may be related to differences in Cit-induced NO production that affects ghrelin levels. However, unlike our results, the effect of Cit on metabolic parameters is higher in females (14).

In the current study, Cit decreased body weight in T2D female rats in a dose-dependent and sex-dependent manner. Body weight-lowering effects of Cit have been previously reported in male rodents (13-17) and can be attributed to Cit-induced decreased food intake. Besides Cit, other NO donors, such as inorganic nitrate, have been shown in a meta-analysis to help reduce body weight (43). Mechanisms underlying the body weight-lowering effects of Cit in female rats include decreased white adipose tissue (WAT), increased brown adipose tissue (BAT) (14), increased lipolysis (44), increased fatty acid β-oxidation (45), and oxidative phosphorylation uncoupling (46).

In our study, the effect of Cit in lowering body weight was dose-dependent, with 1225 mg/kg having the most pronounced effect. Similarly, the improving effect of Cit on the lipid profile in T2D male rats has been reported to be dose-dependent, with the best response obtained at 400 mg/kg (21). In the current study, the effect of Cit in lowering body weight was higher in males than in females. Male mice have greater lean body mass and lose more weight and lean body mass in response to dietary restraint than females; likely, part of this additional reserve is more easily used during food restriction without compromising important physiological functions (47).

The present study demonstrated that Cit causes a dose-dependent reduction in water consumption in T2D rats. We did not find a study to address the effect of Cit on water consumption in T2D. In STZ-induced type 1 diabetic mice, administration of Cit (50 mg/kg/day for 2 weeks) in drinking water decreased water consumption without affecting blood glucose levels compared to untreated diabetic mice (48). A likely explanation for reducing water consumption by Cit includes an NO-dependent increase in renal water reabsorption, as it has been reported that Cit restores NO bioavailability in hypertensive rats (49, 50).

In the current study, Cit at its most effective dose (7 g/L) reduced body weight by 13% in males and 8% in females. This finding has significant implications, as the global rise in obesity represents a serious public health concern, with projections indicating that by 2035, 23% of men and 27% of women worldwide will be living with obesity (1). Most FDA-approved anti-obesity drugs often result in ≤ 10% weight loss and have side effects (5). Therefore, Cit, a non-essential amino acid and a natural compound found in the diet (e.g., watermelon), may serve as a promising alternative to traditional weight-loss medications. However, well-designed clinical trials in humans are needed to confirm its efficacy.

As strengths, our study included both sexes. Approximately only 10% of animal studies in endocrinology involve both male and female subjects, while the majority (66%) exclusively use male animals (51). Additionally, our T2D model (i.e., HFD + low-dose of STZ) imitates the pathophysiology of T2D in humans (24).

As a limitation, we did not assess the underlying mechanisms by which Cit decreases body weight, food intake, and water consumption, including possible changes in leptin and ghrelin as key regulators of feeding and long-term energy homeostasis (52).

### 5.1. Conclusions

The findings of this study showed that Cit decreases body weight, food intake, and water consumption in a sex-dependent (except for water consumption) and dose-dependent manner in obese T2D rats.

ijem-23-3-162367-s001.zip

## Data Availability

The data presented in the study is available on request from the corresponding author during submission or after publication.
