# Supplementary material for: Sex- and Dose-Dependent Effect of L-Citrulline on Body Weight and Food Intake in Obese Type 2 Diabetic Rats
Source: Int J Endocrinol Metab. 2025 Oct 20;23(3):e162367. doi: 10.5812/ijem-162367 (PMC12606883; doi:10.5812/ijem-162367)
Supplement: ijem-23-3-162367-s001.zip [file ijem-23-3-162367-s001.zip › Supplementary Figures.docx]

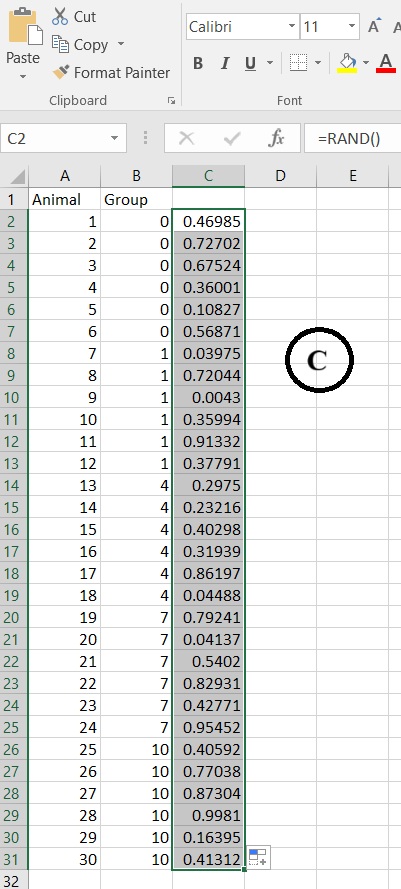

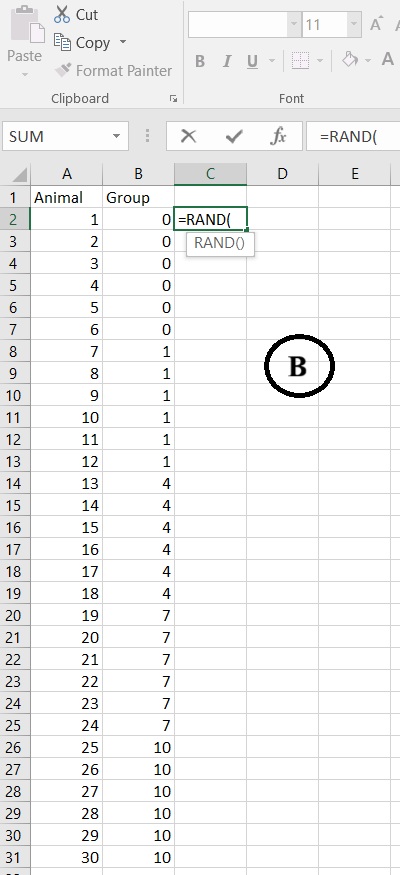

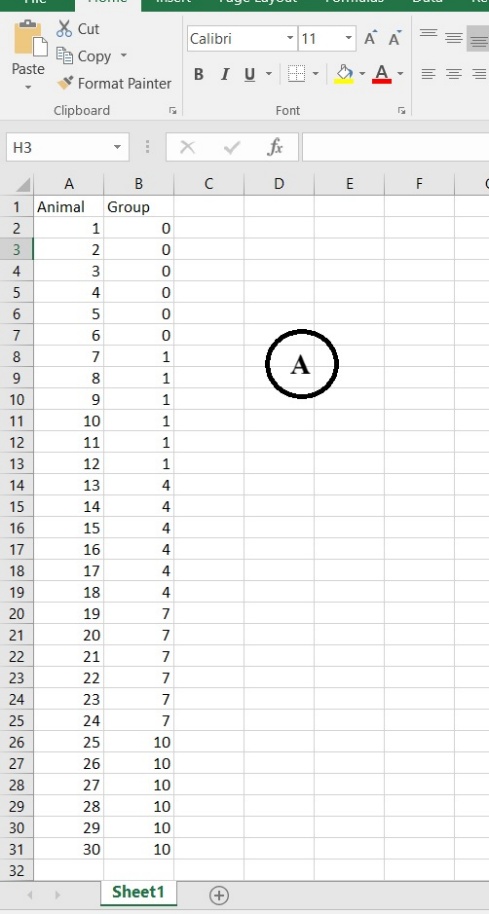


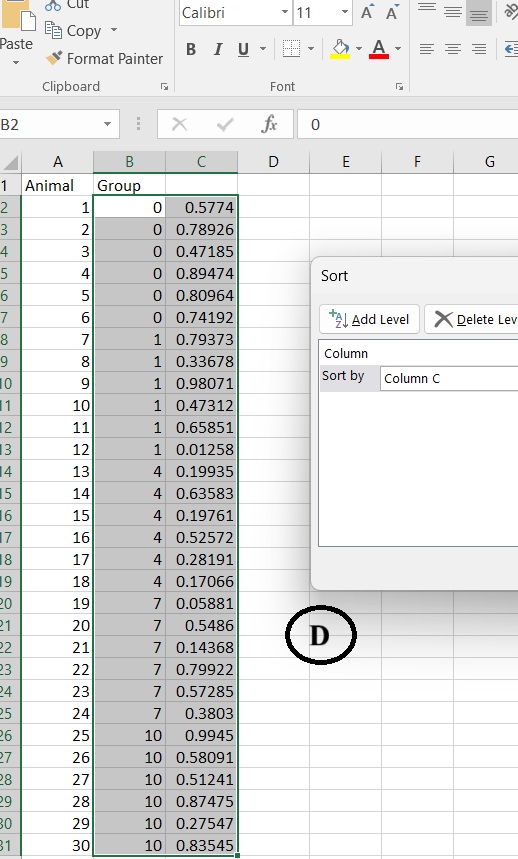


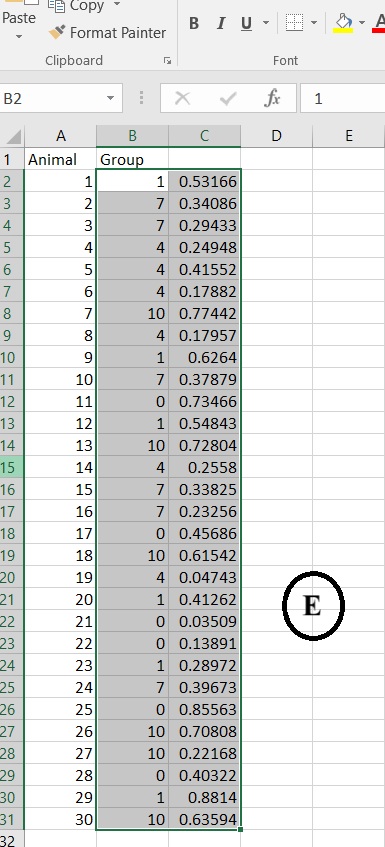


**Supplementary Figure 1.** Randomization of rats using the RAND function in Microsoft Excel (version 13.0). In this study, 30 rats were randomly assigned to five experimental groups (0, 1, 4, 7, and 10), with 6 rats per group, using the RAND function in Microsoft Excel. The randomization process was performed as follows: (A) Animal IDs were entered in column A, and the group labels (in a fixed, non-random order) were listed in column B. (B) In cell C2, the formula =RAND() was used to generate a random number between 0 and 1. (C) The formula was then dragged down from C2 to C31 to generate a column of 30 random numbers. (D) Columns B (group labels) and C (random numbers) were selected. (E) Using the “Sort” function in Excel (Data → Sort), column B was sorted based on the values in column C. This procedure randomly reassigned the group labels to the animal IDs. For example, Rat 1 was assigned to Group 1, Rat 2 to Group 7, and so on (see panel E).

| Cit concentration (µmol/L) | OD_540 nm_ | |
| --- | --- | --- |
|  | Mean | SE |
| 0 | 0.084 | 0.01 |
| 20 | 0.167 | 0.02 |
| 40 | 0.235 | 0.04 |
| 100 | 0.428 | 0.06 |
| 200 | 0.873 | 0.06 |

**Supplementary Figure 2.** Cit standard curve.
